# Partial Remission Without Recurrence in a 9-Year-Old Golden Retriever with Nasal Carcinoma Treated with Prednisolone/Chlorambucil Metronomic Combination Therapy: A Case Report and Literature Review of Molecular Mechanisms

**DOI:** 10.3390/cimb47080660

**Published:** 2025-08-15

**Authors:** Kyuhyung Choi

**Affiliations:** 1 Transdisciplinary Department of Medicine & Advanced Technology, Seoul National University Hospital, Seoul 03080, Republic of Korea; kyudac@snu.ac.kr; 2 Bundang New York Animal Hospital, Seongnam 13637, Republic of Korea

**Keywords:** nasal tumor, nasal carcinoma, prednisolone/chlorambucil metronomic chemotherapy, hyperlipidemia, vascular endothelial growth factor, single cell RNA sequencing

## Abstract

This paper reports the first case in which a hyperlipidemic retriever (due to hypothyroidism) with a nasal tumor was successfully treated—achieving partial remission—and managed using a metronomic combination of chlorambucil (3.74 mg/m^2^, SID) and prednisolone (0.28 mg/kg, SID) orally for 9 months at a general practice. A 35 kg spayed female golden retriever aged 8 years and 8 months with nosebleeds visited the Bundang New York Animal Hospital in July 2023 after being diagnosed with nasal carcinoma. A protocol of 4 weeks of chemotherapy followed by 1 week of rest was repeated in two cycles and continued metronomically for 9 months without pause after the two cycles. The nasal exudate was significantly reduced. The size of the nasal tumor was monitored using computed tomography (CT) imaging at a referral hospital. Since the first occurrence of epistaxis, 18 months have passed (as of January 2025) and the nasal exudate is barely visible, and the vital signs and weight of the dog remain stable. The size of the nasal tumor significantly decreased after 9 months of chemotherapy completion without moderate side effects, and all the blood work was normalized, including hypercholesteremia. This study demonstrates that, in hyperlipidemic cancer patients, a prednisolone/chlorambucil metronomic combination which is cost-effective can be an alternative to tyrosine kinase inhibitors such as sorafenib, even when excluding the price. Through a literature review, the author also investigates the effect of the hyperlipidemic state on cancer, focusing on carcinoma and vascular endothelial growth factor (VEGF), as well as the RAS-RAF-MEK pathway, which is a target for tyrosine kinase inhibitors, in order to reveal the molecular mechanism of chlorambucil metronomic chemotherapy. Also, the author investigates the molecular pathway of carcinoma development in human hyperlipidemia patients through single-cell RNA sequence analysis using open public data, and discusses the molecular action of chlorambucil.

## 1. Introduction

Nasal tumors are often seen in retrievers [1,2]. Radiation therapy is used to debulk the tumor [3,4], and then oral or intravenous chemotherapy is administered [3,5], depending on the prognosis. Anticancer drugs include first-generation chemotherapy, second-generation targeted anticancer drugs, and third-generation immunotherapy drugs [6] [7,8]. Considering their price and effectiveness, first- or second-generation anticancer drugs are mainly used in small-animal practice [9]. In this paper, the author reports a case in which a hyperlipidemic retriever with a nasal tumor was successfully treated and managed using a metronomic [10] combination of chlorambucil (3.74 mg/m^2^, SID) and prednisolone (0.28 mg/kg, SID), which are commonly used in combination for cat nasal tumors [11]. Although this case report only consists of one case due to the retrospective study design, it is significant as it is the first trial of chlorambucil–prednisolone metronomic chemotherapy in a dog. A follow-up case study in the future, involving a normolipidemic retriever with a nasal tumor treated with the same protocol, would enhance the scientific validity. Additionally, the correlation between hyperlipidemia and cancer has been widely studied in humans [12,13], but the detailed mechanism of the hyperlipidemic effect on the growth of cancer is still unclear. The most well-known mechanism regarding cancer growth is that of vascular endothelial growth factor (VEGF) [14], which includes the RAS-RAF-MEK pathway [15], which is essential for targeted anticancer agents. Since the patient in this case study was hyperlipidemic due to concurrent hypothyroidism, the author explains in the following literature review section how chlorambucil works perfectly—not tyrosine kinase inhibitors—in cases of hyperlipidemia, with a focus on the RAS-RAF-MEK pathway.

## 2. Case Description

**Figure 1:**
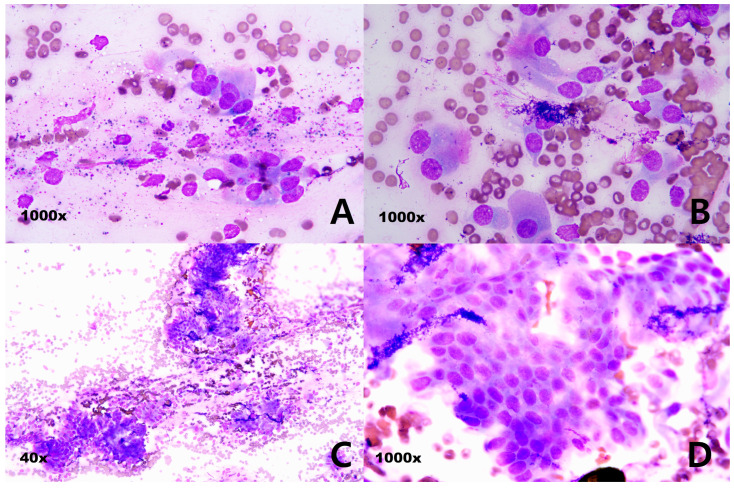
(**A**–**D**) Fine-needle aspiration of nasal mass. Specimen: nasal cavity; single; size: 5 × 2 × 3 cm; incisional. Diff-Quik staining: (**A,B,D**) 1000× magnification, (**C**) 40× magnification. (**A**) Neutrophils can be observed in small numbers. Moderate nuclear anisotropy can be observed, and three small nucleoli can be clearly observed in a thick chromatin pattern. (**B,C**) The cell integrity of the aspirate is very high, and the blood is severely infiltrated. (**B,D**) Various types of nasal epithelial cells, such as columnar ciliated epithelium, columnar epithelium, and goblet cells, can be observed in large and small groups. A basophilic amorphous substance thought to be nasal exudate can be observed, and many melanin granules and eosinophilic granules can be observed in the background. Perinuclear vacuoles, which are mainly observed in squamous cell carcinoma cells, can be observed. (**D**) Cellularity is high and cell shedding is very severe, although cells with a very high degree of malignancy cannot be observed.

In July 2023, a 35 kg neutered female retriever aged 8 years and 8 months visited our hospital with nosebleeds as the main complaint. At the referral hospital, she was first misdiagnosed with immune-mediated thrombocytopenia (IMT) and concurrent hyperlipidemia due to hypothyroidism. There was a severe decrease in platelets (reference range: 16,000/µL, 200,000~460,000/µL), elevation in total cholesterol (450 mg/dL, reference range: 100~330 mg/dL), increased TSH (4.01 ng/mL, reference range: 0~0.5 ng/mL), and decreased T4 (0.5 ng/dL, reference range: 1.3~2.9 ng/dL) in the blood test. The concurrent disease was treated for 4 weeks, and bloodwork results normalized, except for epistaxis, which decreased but persisted. A CT scan was performed to rule out other diseases, and a diagnosis of a newly occurring tumor was given in August 2023. The nasal carcinoma diagnosis was confirmed via consecutive fine-needle aspiration (FNA) (Figure 1A–D). Considering the age of the patient and the highly invasive nature of the procedure, which can lead to a low platelet number due to serious bleeding complications, a histopathological examination was not conducted according to the pet owner’s request. The nasal carcinoma was treated with radiation therapy three times (10 Gy) over 3 days in mid-September 2023. Then, while management with the second-generation anticancer drug sorafenib continued, nasal exudation persisted, and the size of the tumor was not significantly decreased at the referral hospital CT scan. So, the client consented to switching from sorafenib to the first-generation chemotherapy drug combination chlorambucil/prednisolone at the transferred general practice (Bundang New York Animal Hospital) in October 2023. These were given as a combination consisting of 10 mg/day (0.28 mg/kg, SID) of prednisolone and 4 mg/day (3.74 mg/m^2^, SID) of chlorambucil PO. The dose of the medication and selection of the drugs were based on the doctor’s prior empirical experience, demonstrated in [16]. The protocol of 4 weeks of chemotherapy followed by 1 week of rest was repeated, and the nasal exudate decreased significantly. One week of rest was introduced to prevent side effects from the chlorambucil such as bone marrow suppression and gastrointestinal dysfunction. After completing two cycles, the oral chemotherapy was continued metronomically without pause for 9 months without side effects. As of October 2024, chemotherapy was paused after partial remission with no exudate and clinical signs. As of January 2025, 3 months after the chemotherapy completion date, the patient was healthy without any clinical signs, and they remain so as of July 2025, which is 9 months after the metronomic protocol.

**Figure 2:**
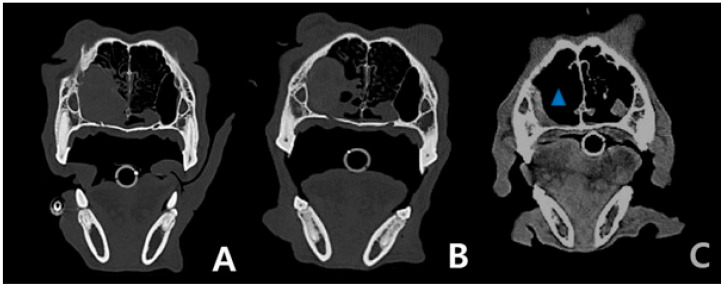
(**A**–**C**) Axial view of the CT scan of the skull. The size of the tumor decreased significantly (blue triangle) after radiation/chemotherapy compared with before radiation/chemotherapy, after radiation, and before chemotherapy.

**Figure 3:**
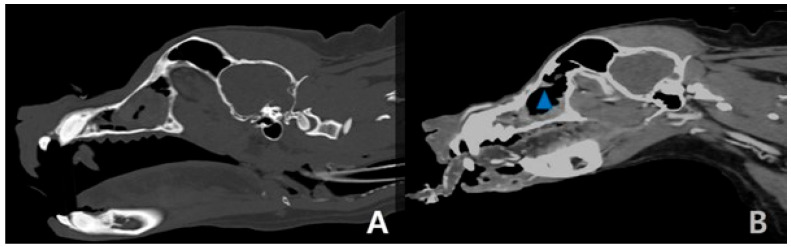
(**A**,**B**) Sagittal view of the CT scan of the skull. The size of the nasal tumor decreased significantly (blue triangle).

A CT scan was performed on a 35 kg spayed female golden retriever aged 8 years and 8 months at a referral hospital (Anonymous A animal hospital) (Figure 2A) in August 2023. Three rounds of radiation therapy over 3 days at 10 Gy were given at the Anonymous B animal hospital, along with a CT scan (Figure 2B and Figure 3A) in September 2023. Fine-needle aspiration (FNA) using the Diff-Quik staining protocol was used by the Green-vet corporation (Yong-in, South Korea) to diagnose the carcinoma (Figure 1A–D) in September 2023 consecutively. All the information including CT scan and FNA results were sent to Bundang New York Animal Hospital, a general practice located in Seongnam, Korea. After a total of 9 months of chemotherapy with chlorambucil (3.74 mg/m^2^; SID; PO) and prednisolone (0.28 mg/kg; SID; PO) at Bundang New York Animal Hospital (general practice), a CT scan was performed (Figure 2C and Figure 3B) for a recheck of the tumor size at the referral hospital (Anonymous C animal hospital) after the first occurrence of the nasal tumor in July 2024. A blood sample was collected from the cephalic vein following 6 h of fasting and centrifuged at 14,500 RPM for 1 min, and the sera were directly measured without dilution using a DRI-CHEM NX500 (Fujifilm, Tokyo, Japan), a dry chemistry analyzer, at room temperature.

Since the radiation therapy and sorafenib treatment was unsuccessful initially, oral administration of methotrexate (2.33 mg/m^2^, SID, PO) and cyclophosphamide (46.72 mg/m^2^, SID, PO) was attempted before chlorambucil treatment, which was based on the doctor’s empirical knowledge but stopped immediately because of a gastrointestinal tract suppression side effect. After these trials, the chlorambucil protocol was introduced and 3 months of the cycle were completed with rest weeks, as previously described (4 weeks of chemotherapy followed by 1 week of rest); the chemotherapy protocol has been continued without rest or pause with a combined metronomic dose of chlorambucil (3.67 mg/m^2^; SID; PO) and prednisolone (0.28 mg/kg, SID, PO) for 6 months. The exudate was significantly reduced. The size of the nasal tumor was significantly decreased after the chemotherapy protocol (Figure 2C and Figure 3B) compared to the radiation-only therapy (Figure 2B and Figure 3A). The nasal exudate has become almost barely visible, and the retriever’s vital signs remain stable with mild palpable lymph node enlargement. Hypothyroidism is being managed by continuously administering levothyroxine sodium, and the retriever’s body weight has increased from 35 kg to 39 kg despite chemotherapy. No other tumor was detected via a recent CT scan except for a liver mass (2.1 × 2.5 cm), which was present before the nasal tumor and was diagnosed as benign via an ultrasound scan. There was no sign of Cushing’s disease despite the frequent use of prednisolone, and the retriever’s appetite was great throughout the entire process.

## 3. Discussion

The combination of prednisolone and chlorambucil to treat nasal tumors is often used in cats [11,17] but not in dogs. This study is the first report of successfully treating a nasal carcinoma in a dog using the combination metronomically. It is known that a high corticosteroid dose decreases the toxicity of Interleukin (IL)-2, which is mainly secreted by activated T cells and facilitates the migration of macrophages by inhibiting migration inhibitory factor [18]. Also, glucocorticoids are empirically used to treat emesis and directly destroy leukemic lymphoblasts [19]. Therefore, steroids are commonly used to treat various types of cancer along with chemotherapy. Chlorambucil induces apoptosis through the alkylation of cross-linked cell DNA strands [20]. In contrast, Sorafenib is a multi-kinase inhibitor (Raf serine/threonine kinases, vascular endothelial growth factor receptor, platelet-derived growth factor receptor-β, and tyrosine kinases) [21]. Therefore, the side effects of chlorambucil may be more significant than those of sorafenib since, theoretically, it may affect not only cancer cells but also normal tissue. Fortunately, the typical side effects of chlorambucil, which include bone marrow suppression (anemia), gastrointestinal signs (vomiting and diarrhea), and alopecia, were not seen in this case. Liver enzymes were mildly elevated during chemotherapy, but other bloodwork results including lipid profile were normal as of 9 months after chemotherapy ((BUN 33.2 mg/dL (9.2~29.2), CRE 0.94 mg/dL (0.4~1.4), ALT 93 U/L (17~78), AST 34 U/L (17~44), and ALP 439 U/L (47~254), total cholesterol 260 mg/dL (127~340), triglyceride 76 mg/dL (21~116)) compared to the pre-chemotherapy bloodwork ((BUN 24.3 mg/dL (9.2~29.2), CRE 0.7 mg/dL (0.4~1.4), ALT 87 U/L (17~78), AST 60 U/L (17~44), and ALP 90 U/L (47~254), total cholesterol 450 mg/dL (127~340), triglyceride 130 mg/dL (21~116)). The slight elevations in ALT (87 to 93) and ALP (90 to 439) are responsible for other factors including aging, diet, and concurrent disease such as hypothyroidism. In the following section, the author investigates research into the molecular mechanism of this successful treatment through a literature review.

In humans, it is reported that tyrosine kinase inhibitors raise triglycerides in dyslipidemia patients [22]. Also, it is known that hypercholesterolemia activates platelets [23] to decrease circulating plasma platelets. Additionally, chlorambucil is effective at lowering cholesterol in human leukemia patients being treated with low-dose metronomic chemotherapy [24].

Although so far it has been not reported in dogs as much as in humans, considering these facts, prescribing chlorambucil rather than tyrosine kinase inhibitors such as toceranib or sorafenib would be more effective in hyperlipidemia patients even if the cost is excluded. In fact, there is a report stating that VEGF/VEGFR inhibitors including tyrosine kinase inhibitors (VEGRF-TKIs) increase the risk of hyperlipidemia [25]. Therefore, this could be the first credible report on the successful management of a canine hyperlipidemic cancer patient with low-dose chlorambucil rather than VEGF/VEGFR inhibitor treatment.

In terms of VEGF, it is critical for the progress of various types of cancers affecting the vascular permeability of tissue and evading the apoptosis of cancer cells [14,26]. Also, it is known that hyperlipidemia can decrease VEGF, leading to impaired angiogenesis [25].

**Figure 4:**
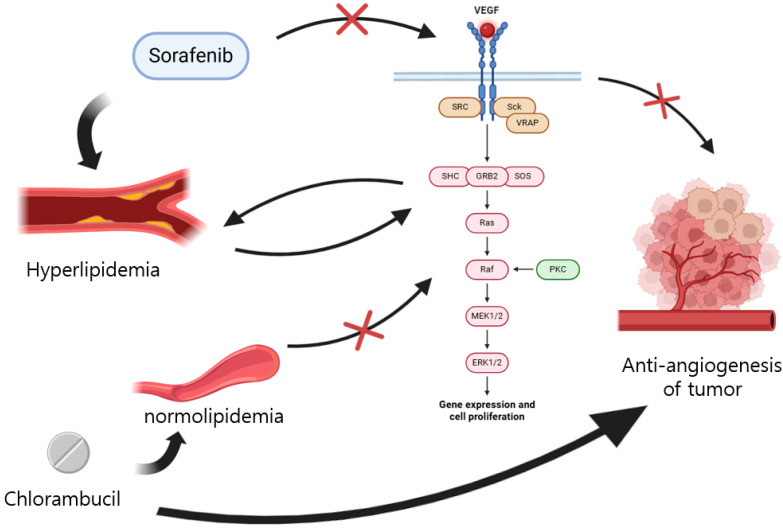
Illustrative mechanism of treating chlorambucil in a hyperlipidemic patient.

The background mechanism of successful cancer treatment in a hyperlipidemic canine patient appears to be, at first, a hyperlipidemic state, before the treatment suppresses tumor angiogenesis via the inhibition of VEGF [27], and then, chlorambucil is introduced to treat nasal carcinoma, improving the hyperlipidemia [24] and platelet elevation [23] without damaging the liver, which is not the same result as would be attained with tyrosine kinase inhibitors [25] such as sorafenib (Figure 4).

As shown in Figure 4, hyperlipidemia and the RAS-RAF-MEK pathway affect each other. Low-density lipoprotein (LDL) is known to be a critical factor in hyperlipidemia [28] and cardiovascular disease [29]. The RAF-MEK-ERK pathway is reported to modulate LDL expression [30], and the LDL receptor was recently revealed to be a key factor of tumor aggressiveness [31]. Moreover, human tumor cells uptake LDL [32], so the use of LDL has been investigated as a carrier for lipophilic chemotherapy including chlorambucil [33,34], although there are limited studies in dogs, except one which elucidated the relationship between lipid profile and hyperlipidemia-related endocrine disease [35].

Additionally, chlorambucil has been used to treat leukemia via affecting the alkylation of target cells [20]. But since it is not target-specific drug, side effects such as bone marrow suppression [36] and gastrointestinal intolerance [37] have been reported in various treatments. Nevertheless, chlorambucil is known to have less hepatotoxicity [38] than other conventional chemotherapy drugs such as methotrexate [39] and azathioprine, the latter of which is also used as an immunosuppressive drug; chlorambucil can also be used to treat biliary cirrhosis [40] in humans. Therefore, chlorambucil has been applied to various types of cancer, such as transitional cell carcinoma in dogs [41] as well as ovarian carcinoma [42] and breast carcinoma [43] in humans. This case report also proves the usefulness of chlorambucil in treating nasal carcinoma in dogs, especially in hyperlipidemic ones.

## 4. Single-Cell RNA Sequencing Analysis

**Figure 5:**
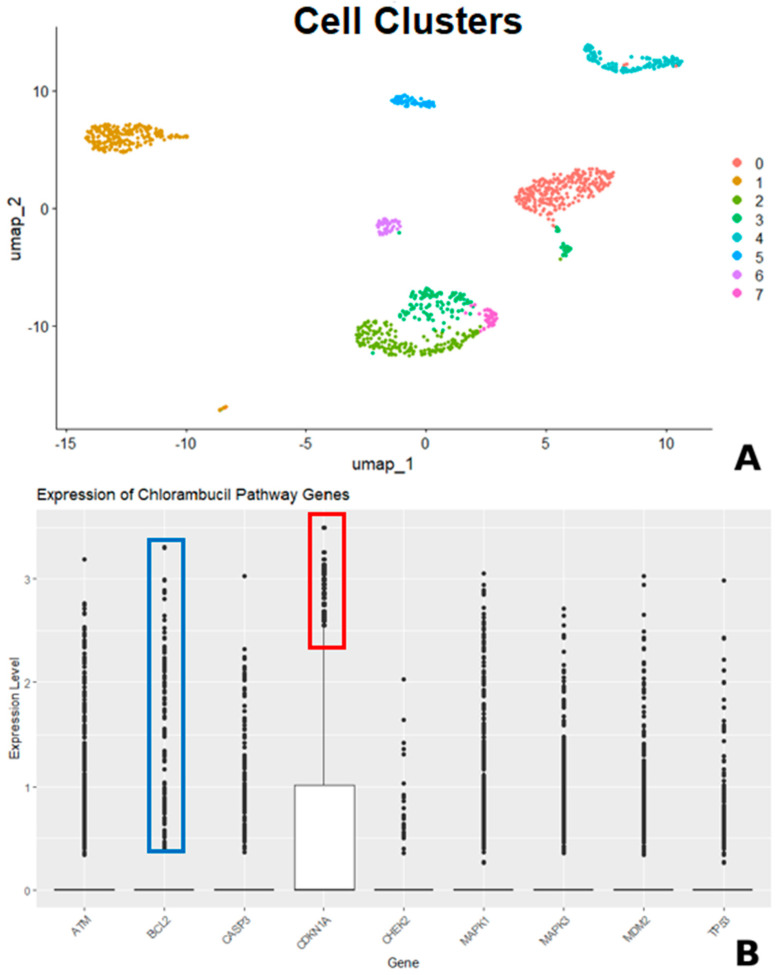
(**A**) Single-cell RNA sequencing analysis of a hyperlipidemic patient and (**B**) expression level of chlorambucil pathway genes.

We can investigate the fundamental mechanism of action of chlorambucil in a hyperlipidemic state through single-cell RNA sequencing analysis using open public data. The GSE197177 dataset, which consists of normal pancreatic human tissue, human pancreatic ductal adenocarcinoma, and human hepatic metastasis tissue, was obtained from the GEO database (https://www.ncbi.nlm.nih.gov/geo/, accessed on 14 March 2025). The author investigated the single-cell RNA sequence of pancreatic ductal adenocarcinoma using R studio and the Seurat package (Figure 5). As previously described, chlorambucil is a DNA-alkylating agent which induces the DNA damage response pathway. This damage stabilizes the tumor suppressor protein p53 via activation of the ATM/ATR signaling cascade [44]. Stabilized p53 translocates to the nucleus and promotes the transcription of target genes, including CDKN1A (p21), a cyclin-dependent kinase inhibitor [45]. p21 inhibits CDK2 and CDK4 complexes, leading to G1/S cell cycle arrest, which allows the cell either to repair DNA damage or to undergo apoptosis [46], and when apoptosis is triggered, this can enhance the cytotoxic effect of chlorambucil, leading to effective treatment responsiveness. BCL2 helps to maintain cell survival by inhibiting apoptosis [47], while responding to DNA damage induced by chlorambucil. Therefore, the relatively higher expression level of CDKN1A than BCL2 (Figure 5, red and blue boxes) may serve as an indicator of chlorambucil’s responsiveness.

## 5. Conclusions

The advantage of chlorambucil is that it is easier to obtain and cheaper than sorafenib. Chemotherapy for large dogs requires a greater quantity of drugs than for small dogs, so many owners hesitate to opt for chemotherapy because of the high cost of treatment. Therefore, chlorambucil/prednisolone metronomic chemotherapy could be an excellent choice for clients who are willing to choose chemotherapy at a general practice rather than a referral hospital because of the high price.

The results of this study also point out that in a hyperlipidemia patient, chlorambucil/prednisolone chemotherapy may be a better option than tyrosine kinase inhibitors because these drugs may aggravate the dyslipidemia of the patient, which was revealed in the background study focused on the RAS-RAF-MEK pathway. However, the author suggests that as this is the first report using chlorambucil in a hyperlipidemic carcinoma patient, more case reports should be accumulated for the drug to be accepted as a standard treatment protocol, considering various factors including age, sex, breed, and other concurrent diseases. Also, the author provides a plausible explanation for chlorambucil’s mechanism of action in a hyperlipidemic cancer patient through single-cell RNA sequencing, which could be further investigated using other open public datasets in follow-up research.

Finally, follow-up research, such as a multicenter trial of chlorambucil in nasal carcinoma patients with or without hyperlipidemia (normolipidemic patients) would strengthen the validation of this case report in the future.

## Data Availability

The raw data supporting the conclusions of this article will be made available by the authors on request.

## Abbreviations

CT	Computed Tomography
IMT	Immune-Mediated Thrombocytopenia
FNA	Fine-Needle Aspiration
VEGF	Vascular Endothelial Growth Factor
